# Ion Channel Density Regulates Switches between Regular and Fast Spiking in Soma but Not in Axons

**DOI:** 10.1371/journal.pcbi.1000753

**Published:** 2010-04-22

**Authors:** Hugo Zeberg, Clas Blomberg, Peter Århem

**Affiliations:** 1 The Nobel Institute for Neurophysiology, Department of Neuroscience, Karolinska Institutet, Stockholm, Sweden; 2 Department of Theoretical Physics, The Royal Institute of Technology, Stockholm, Sweden; École Normale Supérieure, College de France, CNRS, France

## Abstract

The threshold firing frequency of a neuron is a characterizing feature of its dynamical behaviour, in turn determining its role in the oscillatory activity of the brain. Two main types of dynamics have been identified in brain neurons. Type 1 dynamics (regular spiking) shows a continuous relationship between frequency and stimulation current (*f-I_stim_*) and, thus, an arbitrarily low frequency at threshold current; Type 2 (fast spiking) shows a discontinuous *f-I_stim_* relationship and a minimum threshold frequency. In a previous study of a hippocampal neuron model, we demonstrated that its dynamics could be of both Type 1 and Type 2, depending on ion channel density. In the present study we analyse the effect of varying channel density on threshold firing frequency on two well-studied axon membranes, namely the frog myelinated axon and the squid giant axon. Moreover, we analyse the hippocampal neuron model in more detail. The models are all based on voltage-clamp studies, thus comprising experimentally measurable parameters. The choice of analysing effects of channel density modifications is due to their physiological and pharmacological relevance. We show, using bifurcation analysis, that both axon models display exclusively Type 2 dynamics, independently of ion channel density. Nevertheless, both models have a region in the channel-density plane characterized by an N-shaped steady-state current-voltage relationship (a prerequisite for Type 1 dynamics and associated with this type of dynamics in the hippocampal model). In summary, our results suggest that the hippocampal soma and the two axon membranes represent two distinct kinds of membranes; membranes with a channel-density dependent switching between Type 1 and 2 dynamics, and membranes with a channel-density independent dynamics. The difference between the two membrane types suggests functional differences, compatible with a more flexible role of the soma membrane than that of the axon membrane.

## Author Summary

All activity of the brain is manifested in electrical oscillatory patterns, shaped by the firing dynamics of the many neurons forming the brain networks. The underlying mechanisms of the firing pattern in the single neurons are still not fully understood. The distribution and identity of different channel types have been suggested as critical factors. We have suggested that the density of channels in the membrane is a fundamental complementary mechanism. In a hippocampal soma membrane model study we have shown that altering the ion channel densities can cause the membrane to switch between two qualitatively different firing patterns. Here we extend the analysis to two axon membranes. Unexpectedly, both show that channel density alterations do not cause switches between different firing behaviours. We believe that this is an important property of axon membranes, explaining their limited flexibility.

## Introduction

It is now more than 60 years since Alan Hodgkin categorized the firing behaviour in his classical study of isolated axons from the crab *Carcinus maenas* [1]. In many respects his experiments still form the basis for analysis of firing patterns in nervous systems. Using threshold dynamics and maximum frequency as parameters, he identified two major classes of repetitively firing axons (he also defined a class of axons which only fired with difficulty, Class 3): Class 1 axons start firing with very low frequency at threshold stimulation, yielding a continuous *f-I_stim_* relationship, whereas Class 2 axons start firing abruptly with a relatively high frequency (typically 75 Hz) at threshold, yielding a discontinuous *f-I_stim_* relationship.

On the basis of a similar categorization mammalian cortical neurons have also been separated into main classes [2], [3], one exhibiting Class 1 characteristics (regular spiking neurons) and another Class 2 characteristics (fast spiking neurons). The former class consists primarily of pyramidal neurons and the latter primarily of interneurons. This differential classification of excitability has been shown to correlate with a differential bifurcation behaviour of corresponding dynamical models [4]–[6] and successfully been used in analysing the coding properties of neurons [2]–[7]. To avoid confusion, and in accordance with the notation of Tateno and Robinson [7], we in the following use the terms Type 1 and Type 2 dynamics when referring to continuous and discontinuous *f-I_stim_* relationships, respectively. This classification takes the threshold dynamics of the regular and fast spiking neurons, and that of the Class 1 and 2 axons, into account, but not all behavioural aspects of these classes [8].

**Figure 1:**
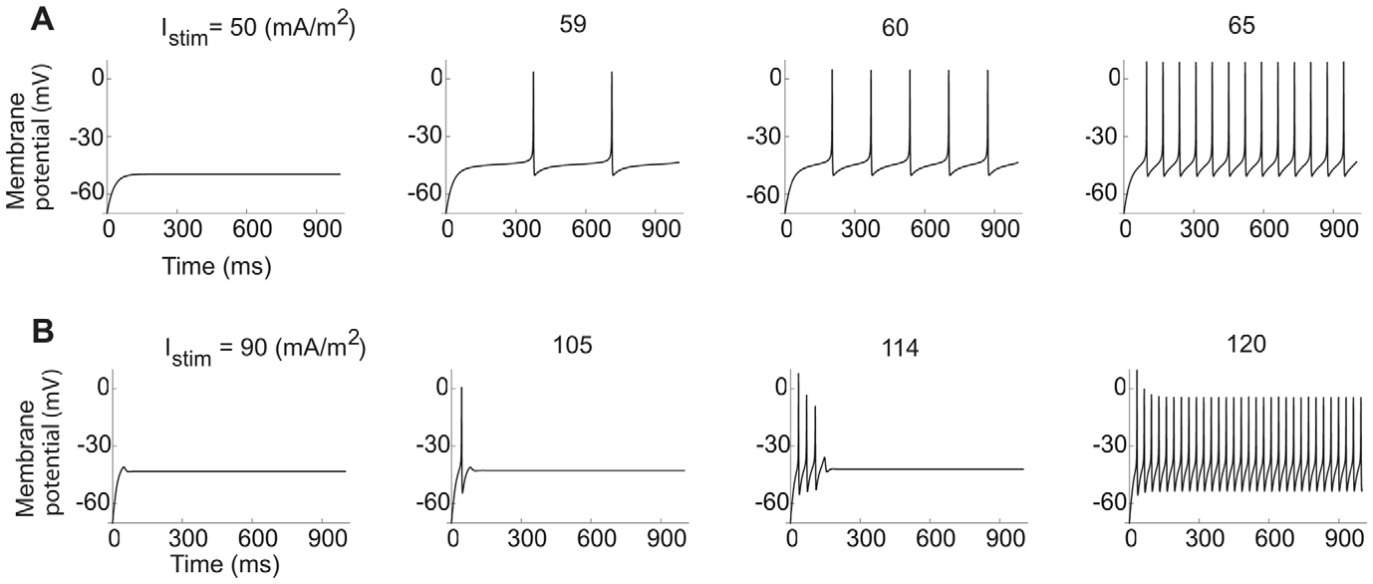
Type 1 and Type 2 dynamics in the hippocampal neuron model. The time-course of the membrane voltage with increasing steady current for low and high K channel densities. (A) 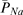 = 20 µm/s and 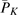 = 5 µm/s. The onset frequency is infinitely small. (B) 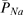 = 20 µm/s and 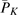 = 5 µm/s. The onset frequency is 30 Hz. Note the damped oscillation with stimulation at 114 mA/m^2^.

The intricate interactions between the many factors involved in the dynamical regulation of neuronal firing are poorly understood [8]. The dominant idea is that different combinations of ion channel types explain the different patterns [9]. In a previous study we proposed a complementary explanation [10], [11]. We showed that both Type 1 and Type 2 behaviour can be simulated in a dynamical model of a hippocampal neuron [12] by varying the membrane density of voltage-gated Na and K channels (i.e. the number of channels per unit of membrane area, reflected in the Na and K permeabilities when all channels are open; see Figure 1 and Methods). The model used was four-dimensional and based on a detailed experimental voltage-clamp study, thus comprising experimentally estimated parameters. The choice of ion channel densities as bifurcation parameters was due to their physiological and pharmacological relevance. Many drugs act by specifically blocking channels and thereby reducing ion channel density both at a somatic and at an axonal level. Perhaps the most used local anaesthetic drug, lidocaine, acts by blocking sodium channels in axons and sensory nerve endings [13]. An increasing number of studies suggest a role for physiological regulation of channel densities, even at a relatively short time scale [14]–[19].

**Figure 2:**
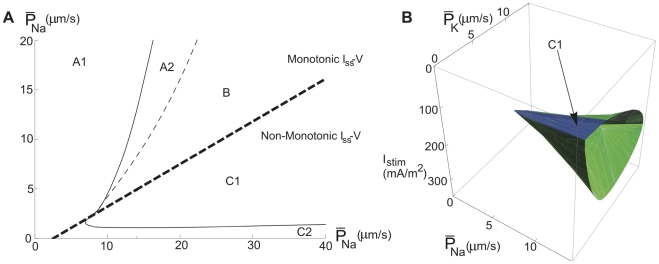
Oscillation maps for the hippocampal neuron model. (A) Regions in the 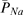
−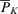 plane associated with different threshold dynamics. Oscillations occur within the area defined by the continuous line. Double-limit cycle bifurcations in the A2 region, Andronov-Hopf bifurcations (together with double-limit cycle bifurcations) in the B region and saddle-node bifurcations in the C1 region. The bold dashed line indicates the border for channel densities associated with three stationary potentials. The map is a projection of a curved plane in the 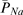
−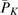
−*I_stim_* space (on which the oscillation starts) to the 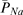
−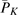 plane. (B) The corresponding three-dimensional map, showing the volume associated with oscillations in the 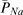
−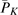
−*I_stim_* space. Oscillations occur in the volume defined by blue and green surfaces. The green surface area represents double-limit cycle bifurcations and the blue area saddle-node bifurcations (SNICs).

Each type of dynamics, i.e., Type 1 and 2, was found to be associated with distinct regions in the channel density plane (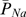
−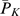) or with corresponding surface areas of an oscillation volume in the 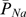
−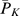
−*I_stim_* space (Figure 2). In regions with high 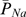 and low 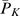 values (region C1) the model exhibits Type 1 dynamics, whereas in regions with higher 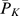 values (regions A2 and B) the model generates Type 2 dynamics.

**Figure 3:**
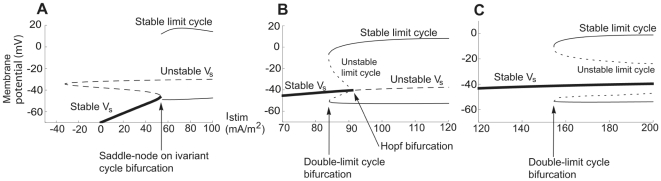
Bifurcation diagrams for the hippocampal neuron model. (A) A saddle node bifurcation in region C1. There are three stationary voltages in the *I_stim_* range of −40 to +50 mA/m^2^. The oscillations occur when the stable stationary potential *V_s1_* merges with a saddle point voltage *V_s2_*. Type 1 threshold dynamics is generated if the limit cycle involves the merged point, i.e. a saddle-node bifurcation on an invariant circle (SNIC). 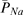 = 20 µm/s, 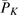 = 2 µm/s. (B) Subcritical Andronov-Hopf and double-limit cycle bifurcations in region B, 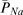 = 20 µm/s, 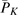 = 10 µm/s. The oscillations emerge at *I_stim_* = 84 mA/m^2^, thus when the corresponding stationary point/voltage still is stable. The loss of stability is due to a double-limit cycle bifurcation, characterized in the variable space by the simultaneous appearance of two limit cycles of opposite stability, one yielding stable and persistent oscillations. This bifurcation is not detectable by the Jacobian matrix of the stationary point; instead the bifurcation depends on the global properties of the variable space. The local Andronov-Hopf bifurcation (also named degenerate Andronov-Hopf bifurcation because of the way the limit cycles collapse onto the equilibrium point [21], [29]) occurs at *I_stim_* = 92 mA/m^2^. There is also an additional Andronov-Hopf bifurcation at higher *I_stim_* (524 mA/m^2^, now shown) that terminates the oscillations. (C) For higher values of 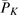 (region A2) these two Andronov-Hopf points collide and disappear (the non-transversal Andronov-Hopf bifurcation), after which no Andronov-Hopf points are present 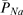 = 20 µm/s, 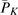 = 20 µm/s.

A bifurcation analysis (see Methods) showed that the Type 1 dynamics of the model is due to saddle-node on invariant circle (SNIC) bifurcations [10], [11]. Figure 3A portrays such a bifurcation in a *V-I_stim_* plot, calculated for the model using region C1 values. The Type 2 dynamics was found to be due to either local Andronov-Hopf bifurcations and/or global double limit cycle bifurcations [10], [11]. The dynamics of the model associated with region B values is due to double limit cycle and subcritical Andronov-Hopf bifurcations (Figure 3B), while the dynamics associated with region A2 is exclusively due to double limit cycle bifurcations (Figure 3C). The double limit cycle bifurcation implies an unstable limit cycle, which is part of a separating structure (sometimes referred to as a separatrix [9], [20]) which separates trajectories turning to a central stable point and those approaching a stable limit cycle.

However, preliminary calculations suggested that the bifurcation structure at the border between regions B (Andronov-Hopf) and C1 (saddle node) is more complex than previously described. When more bifurcation parameters are changed (in our case channel densities and stimulation current) a more intricate loss of stability occurs (e.g. bifurcations with a co-dimension 2) [21].

Thus, to obtain a better understanding of the processes we reanalysed the hippocampal neuron model in more detail. Furthermore, we extended the analysis to two other well-described excitable membranes, i.e., the myelinated axon of *Xenopus laevis* [22] and the giant axon of *Loligo forbesi* [23]. We found that oscillations associated with a subregion of region C1 of the hippocampal model show Type 2 dynamics, and that the oscillations of both axon models exclusively show Type 2 dynamics. We investigated the mathematical background to these findings, using techniques from bifurcation theory. The results suggest that the hippocampal soma and the two studied axon membranes represent two distinct types of membrane with respect to the excitability pattern; membranes with a channel-density dependent switching between Type 1 and 2 dynamics, and membranes with a channel-density independent dynamics. The difference between the two membrane types suggests functional differences, compatible with a more flexible role of the soma membrane than that of the axon membrane.

## Results

### The models

The three membrane models analysed here are all based on voltage-clamp data, and on the formalism originally developed by Hodgkin and Huxley [23] to describe the dynamics of the squid giant axon. The hippocampal neuron model used is that developed by Johansson and Århem [12] to describe small-sized interneurons in hippocampal slices of the rat *Rattus norvegicus*. The myelinated axon model used is basically the same as that developed by Frankenhaeuser and Huxley [22] to describe sciatic nerve fibres from the African clawed frog (*Xenopus laevis*) [24], [25]. The giant axon model used is that of Hodgkin and Huxley [23], describing the dynamics of the giant axon of the squid *Loligo forbesi*.

All the models assume that the membrane current consists of a capacitive current (*I_C_*) and a three-component ionic current (*I_ionic_*), consisting of a Na current (*I_Na_*), a delayed rectifier K current (*I_K_*), and a leak current (*I_leak_*). It should be noted that in all three models the description of the K currents are based on experimentally measured currents, which cannot be regarded as homogeneous, but are most likely the sum of currents passing through several types of voltage-gated K channels. The descriptions of the currents differ slightly between the three membrane types. For instance, the Na and K currents of the squid axon are described using the conductance concept, while the corresponding currents of the myelinated axon and the hippocampal somatic membrane use the permeability concept, developed by Goldman [26] and Hodgkin and Katz [27] (see Methods).

**Figure 4:**
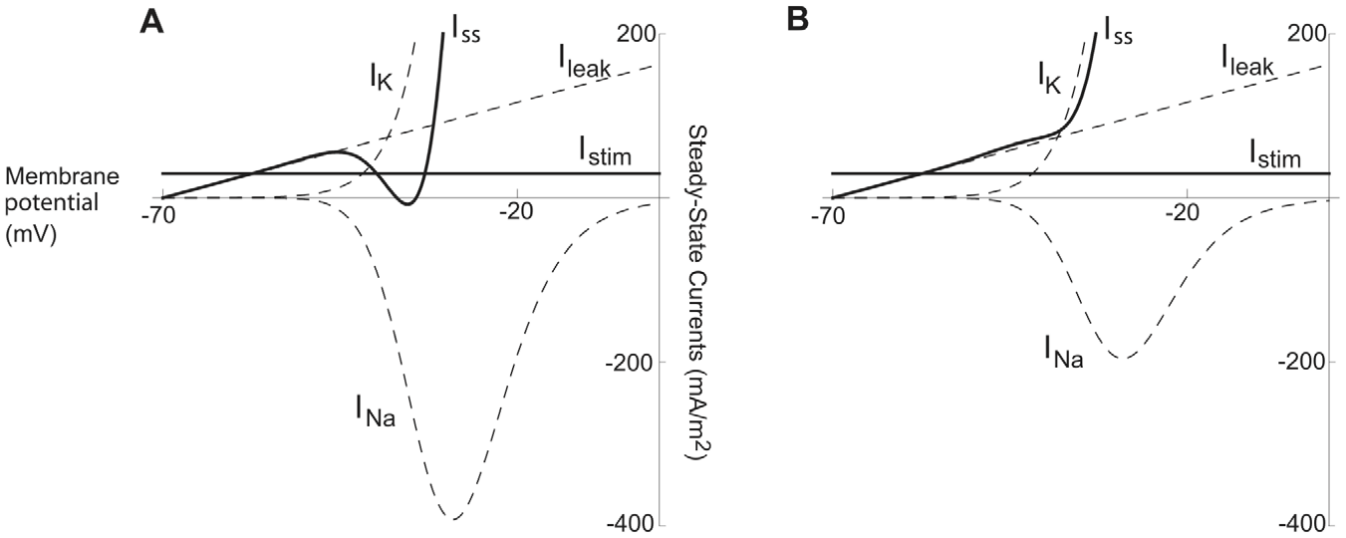
Prerequisites for three stationary potentials (defining region C1). Steady-state currents versus membrane voltage for the hippocampal neuron model. Calculated from Equation 17. The Na channel density is varied while other parameters are maintained constant to demonstrate the requirement of a high Na channel density to obtain three stationary potentials. Inward currents are shown as positive. (A) 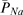 = 30 µm/s and 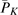 = 5 µm/s. (B) 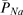 = 11 µm/s and 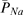 = 5 µm/s.

*I_stim_* is equal to the sum of the capacitive current (*I_C_*), charging the capacitor, and the ionic current *I_ionic_*. Thus,
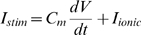
(1)where *C_M_* is the membrane capacitance. To obtain the time-course of *V(t)*, we solve this differential equation numerically, using the expressions for *I_ionic_* presented in the section of Methods. For the analysis of the mathematical nature of the oscillatory activity we determine the stationary potentials (*V_s_*), i.e. the potentials at which the system is in a stationary state and, consequently, the time derivatives of all variables are zero. This was done by solving the following equation for different values of *I_stim_*, 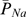 and 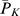 (or 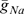 and 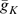 values depending on model; see Methods):
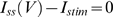
(2)where *I_ss_* is *I_ionic_* at steady state. The stability of the system in the neighbourhood of the stationary potentials was examined by a linearization procedure as described in Methods. Graphical solutions to Equation 2 are presented in Figure 4. A more detailed version of Equation 2 is given by Equation 17 in Methods.

As shown previously [4], [5], [10], [11], some region in the 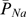
−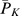 (or 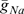
−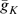) plane must be associated with a non-monotonic *I_ss_-V* curve for the model to produce Type 1 dynamics when entering the oscillatory regime. This is due to the nature of a saddle-node bifurcation on an invariant circle (SNIC), requiring an *I_stim_* interval at which Equation 2 yields three solutions (i.e. *V_s1_*, *V_s2_* and *V_s3_*; see Methods). Thus, Type 1 threshold dynamics occurs only when 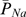 (or 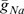) is of a relatively large magnitude, giving the *I_ss_-V* curve a non-monotonic, N-like shape (Figure 4). Hence, a switch from Type 1 to Type 2 firing dynamics takes place when 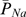 (or 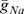) is reduced (and 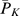 or 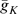 remains intact), corresponding to Na channels being blocked. It should here noted that the existence of three stationary solutions of Equation 2 does not guarantee Type 1 dynamics, as has been pointed out previously [5] and will be seen in the following.

### The hippocampal model: Type 1 and 2 dynamics

**Figure 5:**
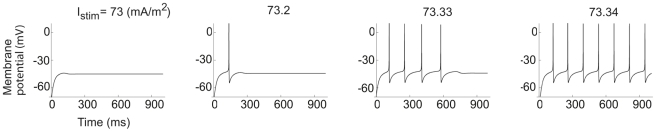
Type 2 dynamics within region C1 for the hippocampal neuron model. The time-course of the membrane voltage with increasing steady current. 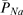 = 40 µm/s and 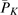 = 15 µm/s. The onset frequency is 8 Hz.

**Figure 6:**
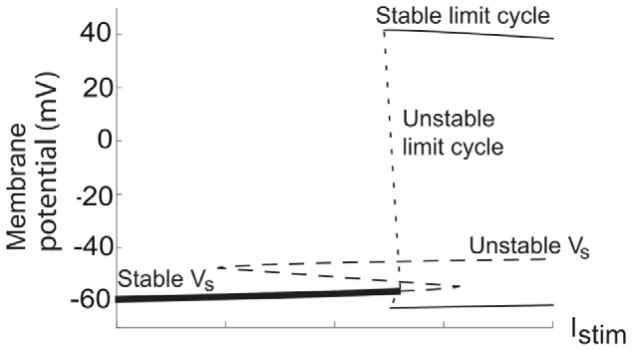
A Andronov-Hopf bifurcation within region C1. Schematic bifurcation diagram showing a subcritical Andronov-Hopf bifurcation within the range of three stationary potentials. The distance between the Andronov-Hopf bifurcation and the coalescence of *V_s1_* and *V_s2_* has been extrapolated.

In our previous examination of the hippocampal model (see Figure 2), we defined the C area as the region where the model shows a non-monotonic *I_ss_-V* relationship (and Equation 2 yields three stationary potentials at some *I_stim_*), with area C1 representing the subregion associated with oscillations. As mentioned above, most of this region is associated with Type 1 threshold dynamics. A more detailed analysis reveals, however, that for density values close to the border of the B area the model demonstrates Type 2 dynamics (Figure 5). This can be shown to be due to an Andronov-Hopf bifurcation when the most negative stationary potential (*V_s1_*) becomes unstable as indicated in the bifurcation diagram of Figure 6 (cf. Figure 3A).

**Figure 7:**
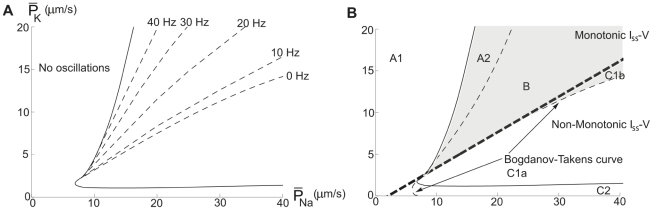
Revised oscillation maps for the hippocampal neuron model. Regions associated with oscillations in the 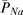
−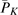 plane, showing the existence of Type 2 dynamics within region C1. (A) Onset frequencies. (B) Oscillation map for comparison with the map of Fig. 2, showing the subregions C1a and C1b. The border between C1a and C1b closely follows the Bogdanov-Takens bifurcation curve (see Table 1).

Figure 7A depicts an oscillation map in the channel density plane, on which minimum frequencies are indicated. As seen on Figure 7B, the line delineating zero frequency deviates from the C1 border at 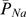 = 14 µm/s and forms a separate, narrow region below this border at higher densities of the Na channel. Below we will denote this subregion C1b and the remaining, larger, region of C1, associated with Type 1 behaviour, C1a. The oscillation map revised accordingly is shown in Figure 7B, where the border between C1a and C1b represents a curve in the ion channel density plane at which Bogdanov-Takens bifurcations occur [28], [29] (see Methods and Table 1). (For the role of Bogdanov-Takens bifurcations in the Hodgkin-Huxley model, integrate-and-fire models and the Morris-Lecar model, see [30]–[33].)

**Table 1:** Characterization of Regions in the Channel-density Plane of the Models.

Region	Oscillations	Bifurcation type	Discontinuous *f-I_stim_* curve	Continuous *f-I_stim_* curve	Three *V_s_* for near-threshold stimulation
A1	No				
A2	Yes	Double limit cycle	Yes		No
B	Yes	Subcritical Andronov-Hopf or double limit cycle	Yes		No
C1a	Yes	Saddle-node		Yes	Yes
C1b	Yes	Subcritical Andronov-Hopf or double limit cycle	Yes		Yes
C2	No				Yes

The channel-density plane corresponds to the permeability or conductance (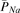
−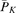 or 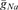
−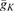) plane. The bifurcation type refers to the onset of oscillations. *V_s_* is the stationary potential.

It should be noted that in addition to the C1b region, there is another C1 subregion, a narrow strip along the borders to the B, A1 and C2 regions for 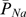 values below 14 µm/s, associated with a saddle-node bifurcation that causes Type 2 dynamics (non-SNIC), CIc. However, for reasons that will become clear from the analysis of the behaviour of axonal membranes, we will here focus on the dynamics associated with the C1b region. A summary of the regions in the density plane is given in Table 1.

**Figure 8:**
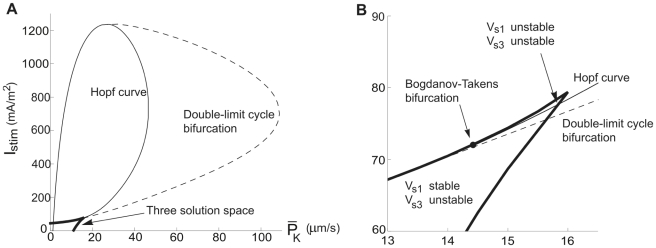
Bifurcation curves and the three-root solution space for the hippocampal neuron model. *I_stim_*−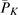 diagrams at 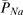 = 40 µm/s. The thick continuous line defines the region associated with three-root solutions of Equation 17. The thin continuous line is the Andronov-Hopf bifurcation curve and the hatched line, defining the oscillation limit, is the double limit cycle bifurcation curve. (A) An overall perspective. (B) A detailed view of the cusp of the three-root solution space to describe the two subregions, defined by the stability of the stationary potentials. The Bogdanov-Takens bifurcation point is marked.

The explanation for the deviant (i.e. Type 2) threshold dynamics in the region associated with non-monotonic *I_ss_-V* curves (region C1b) becomes evident when the corresponding *I_stim_*−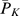 diagrams are considered (similar bifurcation diagrams have been successfully used to analyse comparable models [34]). Figure 8 illustrates two such diagrams for 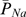 = 40 µm/s, one overview and another highlighting the structure at the cusp of the three-root region (which is part of the non-monotonic *I_ss_-V* region), with thick continuous lines. The thin continuous line marks points associated with Andronov-Hopf bifurcation dynamics and the hatched line depicts the double-limit cycle bifurcation. The Andronov-Hopf bifurcation line intersects the three-solution region and collides with the saddle-node bifurcation line in a Bodganov-Takens bifurcation point [29]. Hence, at the cusp of the three-root region, the Andronov-Hopf line forms a small subregion, characterised by unstable *V_s1_* and *V_s3_*. Thus, at these permeabilities and stimulation currents the threshold dynamics is due to subcritical Andronov-Hopf bifurcations and not to saddle-node bifurcations, and the model will show typical Type 2 behaviour with a minimum non-zero threshold frequency.

### Axonal models: Exclusively Type 2 dynamics

How general is our present description of neuronal models? And how does the density of channels influence the threshold dynamics and firing patterns in other models? To address these issues, two well-described excitable membranes, i.e., the node in myelinated axons of *Xenopus leavis* [22] and the giant axon of the squid *Loligo forbesi* [23] were examined. Both these membranes are similar to the hippocampal membrane with reference to channel composition and kinetics (see Table 2), as can be inferred from the similar mathematical formalism used (see Methods). Nevertheless, the dynamics of both axon membranes were found to show principal differences from that of the hippocampal neuron membrane.

**Table 2:** Kinetic Parameter Values for the Models.

Model	I_Na_ activation *(m^3^, m^2^)*		I_Na_ inactivation *(h)*		I_K_ activation *(n^4^,n^2^)*		Leak conductance
	V_1/2_ (mV)	s (mV)	V_1/2_ (mV)	s (mV)	V_1/2_ (mV)	s (mV)	*g_L_* (S/m^2^)
Hippocampal neuron	−11	10	−48	−5	−8	5	2.3
Myelinated axon	−26	10	−63	−5	−32	5	303
Squid axon	−22	8	−57	−7	−15	20	10

#### The myelinated axon model

**Figure 9:**
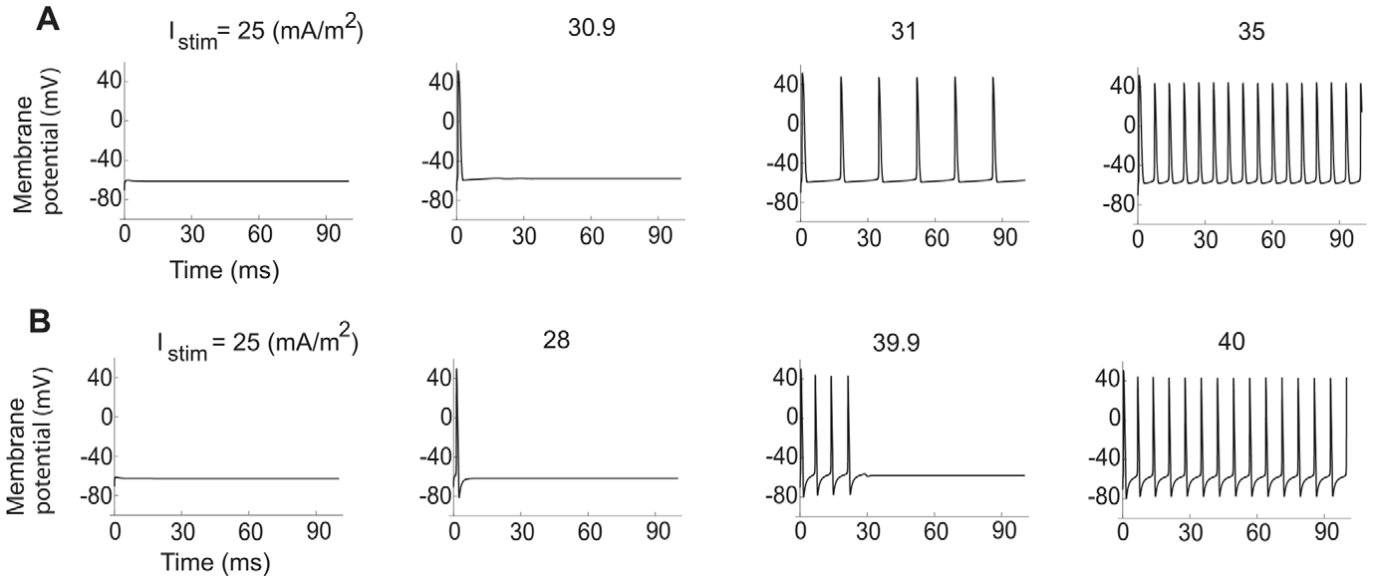
Exclusively Type 2 dynamics in the myelinated axon model. The time-course of the membrane voltage with increasing steady current for low and high K channel densities. (A) 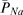 = 300 µm/s and 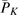 = 0 µm/s. The onset frequency is 59Hz. (B) 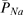 = 300 µm/s and 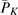 = 40 µm/s. The onset frequency is 139Hz.

Figure 9 documents the calculated time-course of the changes in voltage calculated to occur in the model of the myelinated axon at different stimulation amplitudes for low and high densities of K channel (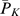 = 0 and 40 µm/s). In this case, no combination of channel densities yielded Type 1 dynamics. However, the shape of the individual spikes differed at low and high K channel densities, an afterhyperpolarization being prominent only at a high K channel density. As shown in the figure, this model, in contrast to the hippocampal model, displays repetitive firing at 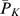 = 0, which may reflect the fact that myelinated axons at a later evolutionary stage, i.e. mammals, lack K channels and still fire repetitively [35].

**Figure 10:**
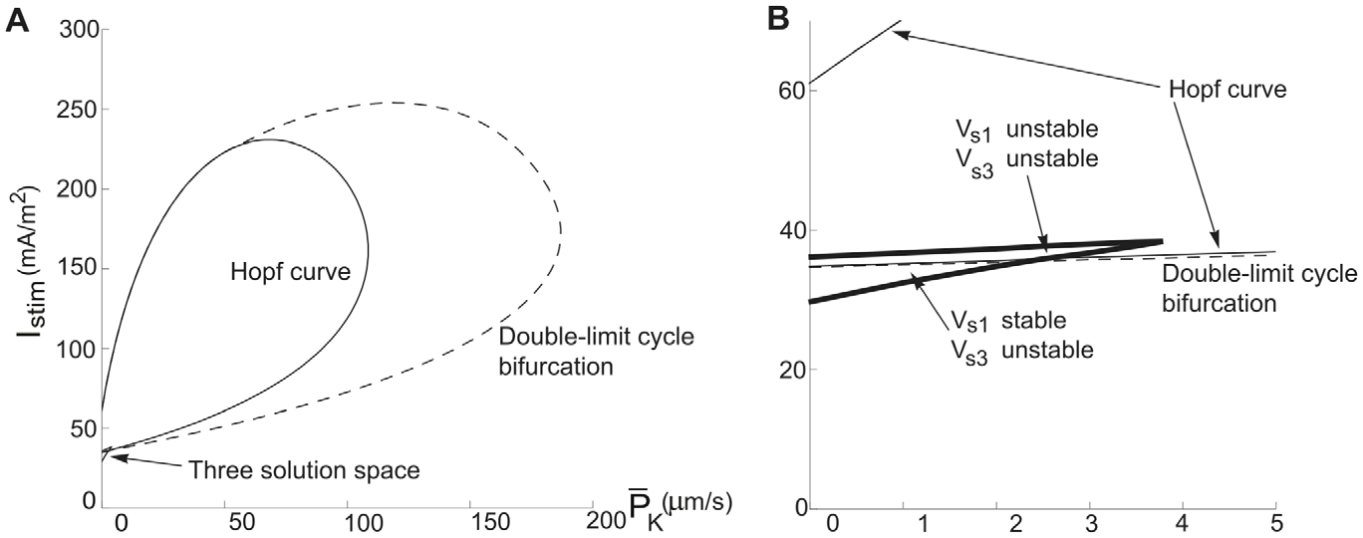
Bifurcation curves and the three-root solution space for the myelinated axon model. *I_stim_*−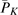 diagrams at 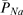 = 200 µm/s. The thick continuous line defines the region associated with three-root solutions of Equation 14. The thin continuous line is the Andronov-Hopf bifurcation curve and the hatched line is the double limit cycle bifurcation curve. (A) An overall perspective. (B) A detailed view of the cusp of the three-root solution space to describe the subregions.

A stability analysis revealed the mechanisms involved. Figure 10 displays *I_stim_*−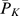 diagrams, showing an Andronov-Hopf bifurcation line within the three-solution space. As summarised below, the resulting channel-density map shows a region with non-monotonic *I_ss_-V* relationship. However, unlike the narrow Andronov-Hopf region C1b of the hippocampal neuron model, the corresponding region of the myelinated axon model covers the whole C1 area. Consequently, saddle-node bifurcation dynamics is missing, explaining the absence of Type 1 dynamics in the myelinated axon model. It can also be noted that the double-limit cycle bifurcation occurs very close to the Andronov-Hopf bifurcation, why great care is needed to distinguish them numerically. That this kind of change can occur very close to each other in a parameter space is a known feature in these kinds of models [34], [36].

The findings suggest less dynamic flexibility in this axon membrane than in the hippocampal neuron model discussed above. Since the hippocampal neuron model is based on measurements of the soma membrane properties, the comparison between the myelinated axon and the hippocampal model mainly concerns a comparison between axonal and soma membranes. To get further information about the functional relevance of the found differences between axon and soma membranes we next analysed the classical squid giant axon membrane, using the description given by Hodgkin and Huxley [23].

#### The squid axon model

**Figure 11:**
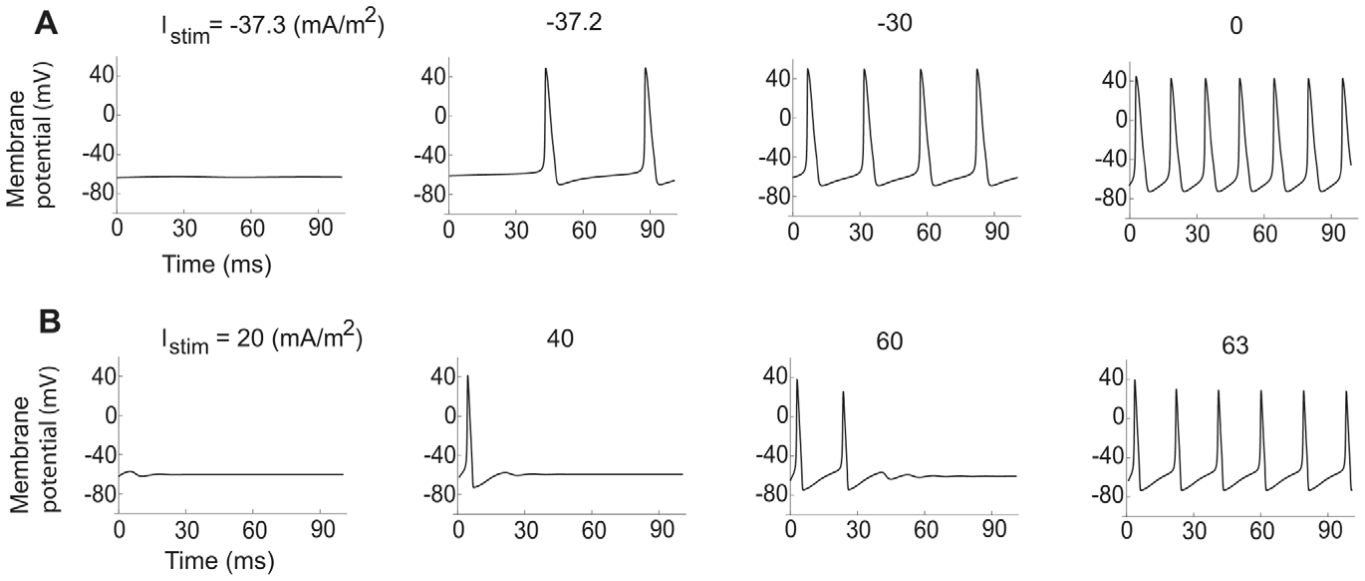
Exclusively Type 2 dynamics in the squid axon model. The time-course of the membrane voltage with increasing steady current for low and high K channel densities. (A) 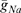 = 1200 S/m^2^ and 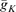 = 50 S/m^2^. Onset frequency is 22 Hz. (B) 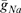 = 1200 S/m^2^ and 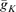 = 360 S/m^2^ (values used by Hodgkin and Huxley in their original study from 1952 [23]). The onset frequency is 52 Hz.

Calculations showed that the giant squid axon model in similarity with the myelinated axon did not show saddle-node or Type 1 dynamics (Figure 11). In contrast to the myelinated axon model, however, the squid axon model could not fire repetitively at zero K channel density (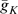 = 0), possibly related to the much higher leak conductance in the myelinated axon (see Table 2). The firing pattern at low 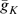 values differed from that at higher 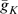 values; it never showed damped oscillations.

**Figure 12:**
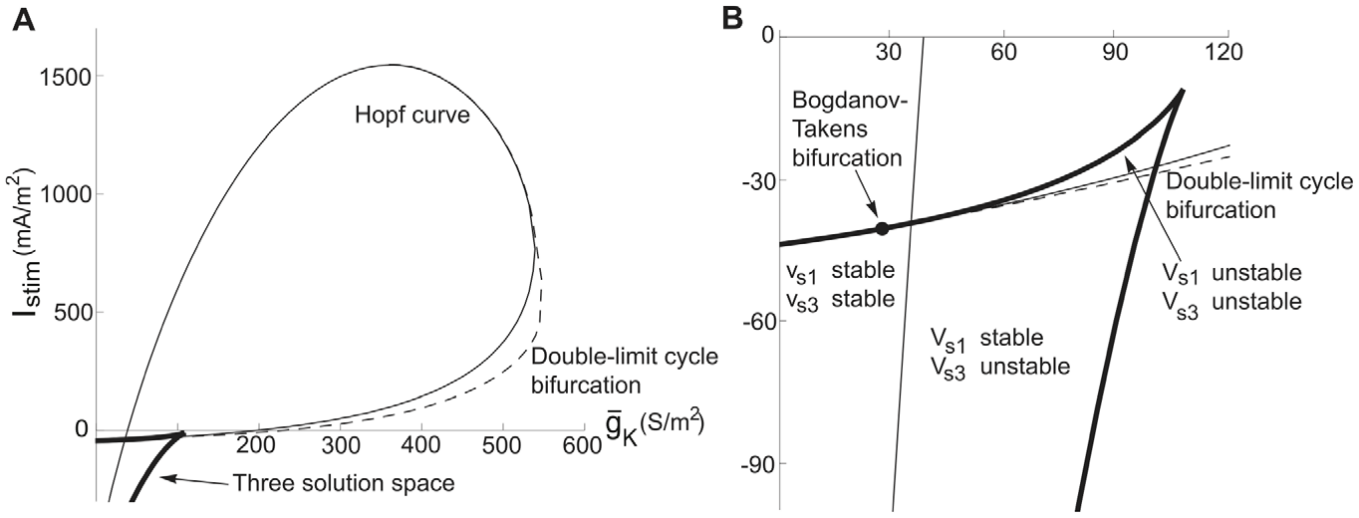
Bifurcation curves and the three-root solution space for the myelinated axon model. *I_stim_*−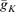 diagrams at 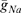 = 1200 S/m^2^. The thick continuous line defines the region associated with three-root solutions of Equation 14. The thin continuous line is the Andronov-Hopf bifurcation curve and the hatched line is the double limit cycle bifurcation curve. (A) An overall perspective. (B) A detailed view of the cusp of the three-root solution space to describe the three subregions. The Bogdanov-Takens bifurcation point is marked.

**Figure 13:**
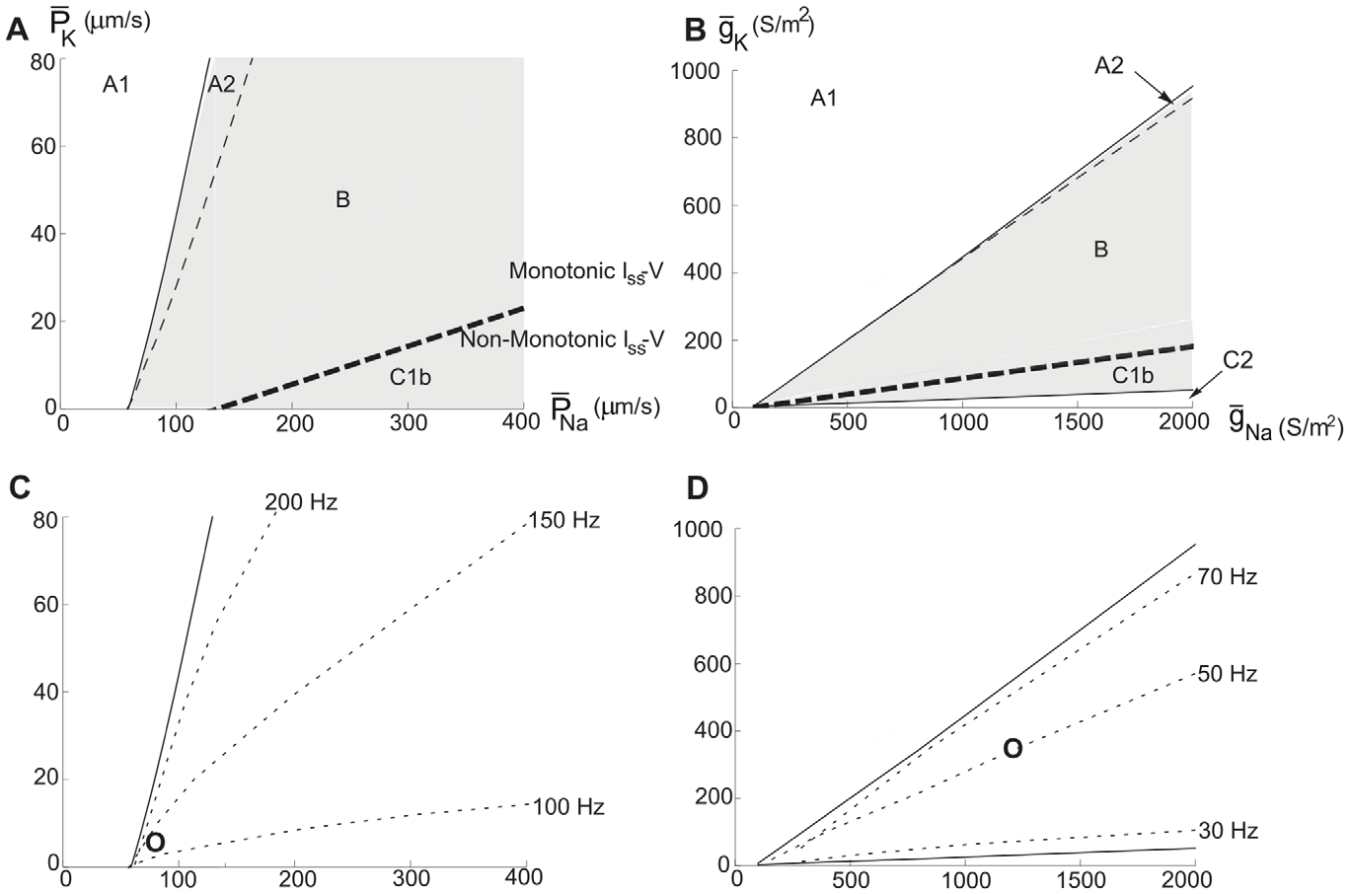
Oscillation maps for the axon models. Regions associated with oscillations in the 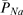
−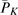 or 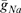
−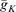 plane. (A) The frog myelinated axon model. (B) The squid giant axon model. As seen there is no C1a region in any of the maps and consequently both axon models lack Type 1 dynamics. Note also that the myelinated axon model (A) allows oscillations for 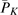 = 0 (no K channels). (C) Onset frequency in the myelinated axon model. (D) Onset frequency in the squid axon model. Circles indicates the original values used by Hodgkin and Huxley for the model of the axon of *Loligo forbesi* [23] and Frankenhaeuser and Huxley for model of the sciatic nerve of *Xenpus leavis* [22].

The reason for the absence of saddle-node dynamics is evident in the *I_stim_*−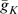 diagram of Figure 12. As seen, there exists a three-root region, but in similarity with the myelinated axon model, there is an Andronov-Hopf bifurcation line within this area. Figure 13 shows the oscillation map for the squid axon model (B) in comparison with that of the myelinated axon (A). Figure 13 also shows the onset frequencies of the two axon models, being typically higher than 30 Hz for the squid axon (D) and higher than 100 Hz for the myelinated frog axon (C). In summary, the present analysis suggests a similar response pattern for models of different axon membranes in spite of considerable differences in mathematical structure. Furthermore, it suggests a functional difference between axon and soma membranes, the latter being more flexible.

## Discussion

The manner in which interactions between the ionic currents in a neuron determine the pattern and dynamics of firing is a multifaceted problem, having many ramifications within theoretical systems biology [8]. In a previous study, we demonstrated that altering the ion channel densities in a four-dimensional dynamical model (comprising experimentally measurable parameters) of a hippocampal neuron could cause switches between Type 1 and Type 2 firing behaviour [10], [11]. We also suggested that this channel density paradigm may explain the different threshold dynamics of regular and fast spiking cortical neurons [2], [7], [8], as well as that of Class 1 and Class 2 axons in *Carcinus maenas* [1]. The physiological and pharmacological relevance of channel densities as bifurcation variable has recently been experimentally confirmed [37], [38].

In the present analysis we show that corresponding channel density alterations in two well-studied axon models (the amphibian myelinated axon and the squid giant axon) cannot change the firing dynamics, exclusively being of Type 2. This suggests that the hippocampal soma and the two axon membranes represent two distinct types of membrane with respect to the excitability pattern; one more flexible that can switch channel-density dependently between Type 1 and Type 2 dynamics (represented by the hippocampal neuron membrane and for simplicity here denoted M1/2) and one, less flexible, that exclusively shows Type 2 dynamics (represented by the membranes of the two axons and here denoted M2).

The mathematical background to the flexibility of the first membrane type is the existence of two types of bifurcations associated with separate regions in the 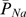
−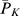 plane (regions B and C1). Type 1 dynamics (region C1) is associated with saddle-node on invariant cycle bifurcations (SNIC) and Type 2 dynamics (region B and A2) with double-limit cycle bifurcations (in some cases along with a subcritical Andronov-Hopf bifurcation). A requirement for the occurrence of a saddle-node bifurcation is the existence of three stationary voltages at near threshold stimulation and thus, a non-monotonic *I_ss_-V* relationship. In the present analysis we show that this requirement is not sufficient. In all three models we find regions in the 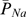
−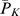 plane with a non-monotonic *I_ss_-V* relationship, that are associated with Type 2 dynamics (C1b regions). In the hippocampal neuron model the region consists of a narrow band, and in the axon models they cover the whole C1 region, and thus, the axonal models lack Type 1 threshold dynamics (for the analysis of the bifurcation structures in regions with non-monotonic *I_ss_-V* relationships of generic two-dimensional models, see [30]–[33], [39]).

### Functional implications: The plastic soma membrane and the restricted axon membrane

What is the functional reason for the difference in the flexibility of threshold dynamics between the two membrane types defined above (M1/2 and M2, represented by membranes of the hippocampal neuron and the axons)? Type 1 and 2 dynamics per se most likely have different functional roles; Type 1 dynamics being required for low frequency firing and Type 2 being essential for doublet spiking (in hippocampal interneurons, [40]; in dorsal horn neurons, [41]). and for synchronization of firing in coupled neurons (in the synchronization case due to the fact that both a phase advance and a phase delay are possible; the phase response curve being predominantly positive when the oscillations appear via a saddle-node on invariant cycle bifurcation, but both negative and positive in the case of the Andronov-Hopf bifurcation; see [42]). But what about the difference in flexibility between the two membrane types presently discussed?

The membranes of the soma and the proximal portion of the axon, which most likely determine the dynamics of the hippocampal neurons analysed here, can be assumed to show a considerable flexibility in their roles as integrative summing points, requiring (transmitter- or trafficking-) induced switchability between Type 1 and Type 2 dynamics (see e.g. [43], [44]). Such flexibility has also been shown in cortical fast spiking (Type 2) interneurons [45]; Type 2 dynamics being changed to Type 1 dynamics when the K channel density (in soma) is reduced in dynamic clamp experiments. Similarly, fast spiking mesenchaplic V neurons have been shown to belong to the M1/2 class [37].

In contrast to the flexible or plastic soma membrane, the axon membranes form passive information transport chains, requiring reliable triggering mechanisms (i.e. high current thresholds leading to rejection of low stimulus noise, and temporal all-or-none responses, meaning that the first spike always occurs early at threshold stimulation) and, therefore, Type 2 dynamics. It should be pointed out, however, that the main value of the axon type dynamics most likely relates to its action when associated with a trigger zone, which likely is the case of the distal process of the dorsal root ganglion. Features associated with Type 2 dynamics, such as subthreshold oscillations and doublet spiking have been postulated to play an important role in pain modulation [46], [47]. Thus, the Type 2 nature of the axon plays a role both in the information propagation and modulation.

Clearly, this discussion, based on an analysis of axons from one amphibian (*Xenopus laevis*) and one cephalopod (*Loligo forbesi*), cannot be generalized to axons from all animal phyla. As mentioned above, certain axons from the arthropod *Carcinus maenas* display Type 1 dynamics [1], [23], suggesting that that their cell membranes are of M1/2 type (for a computational analysis of modifying the dynamics of squid axons, see [48]). What about vertebrate axons in general? The phylogenetic bifurcation between the vertebrate and the arthropod lines occurred more than 500 million years ago, allowing a considerable time for specialization of axon membranes. To get information on this issue, we used the data from a voltage-clamp analysis of myelinated rat axons by Brismar [35] to construct and evaluate a dynamical model. The computations suggest that the rat myelinated axon membrane is of M2 type, exclusively displaying Type 2 dynamics (Figure S1 and Text S1). In conclusion, the present analysis shows that axon membranes of two vertebrate and one mollusc species are of M2 type, and axon membranes from one arthropod species are either of M1/2 or of M2 type. More studies are needed to determine whether vertebrate axons mainly are of M2 type or not. It should here be noted that the phylogenetic distance between present day molluscs and arthropods is considerably shorter than that between present day vertebrates and molluscs or arthropods.

### Structural implications: Different trigger zones in cortical pyramidal cells and interneurons?

Mammalian cortical pyramidal cells have been shown to display both Type 1 (regular spiking) and Type 2 dynamics (fast spiking), with Type 1 in majority [7], [44]. Assuming that the trigger zone dynamics is of critical importance for the dynamics of the neuron in toto, the present analysis suggests that the membrane of the trigger zone of the majority of pyramidal cells is of M1/2 type. This also suggests that the assumed trigger zone of pyramidal cells, the initial segment of the axon [49], [50], is not formed by a M2 membrane, contrary to the presently studied axons. A way to experimentally test the hypothesis of a M1/2 membrane as trigger zone in pyramidal cells could be to analyse the results of introducing K channels with the dynamic clamp technique. Such a test is under way.

Contrary to the majority of pyramidal cells, mammalian cortical interneurons mainly display Type 2 dynamics (fast spiking). This suggests that the membranes of their trigger zones are either of M1/2 or of M2 type. In the latter case the trigger zone could be assumed to be located in the axon proper; i.e. in the first node of Ranvier or in an initial segment that is more functionally (and structurally?) axon-like than that of the pyramidal cells. A way to experimentally separate between these two hypotheses (whether the trigger zone in interneurons is of M1/2 or M2 type) could be to analyse the dynamics after blocking K channels. Such a test is also under way.

### Pharmacological implications: Network effects of switches between Type 1 and 2 dynamics

As pointed out previously [10], the possibility to modify the threshold dynamics of neurons suggests novel scenarios for the action of channel active drugs such as general anaesthetics; implying mechanisms where selective blocking ion channels in critical neurons induces a switch from one brain state (e.g. associated with consciousness) characterized by certain frequency patterns to another state (e.g. associated with general anaesthesia) characterized by other frequency patterns. Network modelling has shown that such ideas are feasible. Thus, selectively blocking K channels in critical inhibitory neurons (assuming M1/2 membrane trigger zones) in a network of excitatory and inhibitory neurons, distance-dependently connected, can lead to switches from unsynchronised high frequency to synchronised low frequency mean network oscillations [39]. The mechanisms of synchronisation at the network level are still not well understood, but the mechanisms at a cellular level have been extensively studied and a tight connection between the bifurcational structure and the phase-response curve has been established [44], [51]–[53]. Interneurons with Type 2 dynamics have recently been shown to account for the cortical γ-oscillations (20–80 Hz) [54], which are considered to provide a temporal structure for information processing in the brain [55].

### Reducing and increasing the dimensionality of the models: Chaos

Since two of the eigenvalues always are real and negative in the models here discussed, it suggests that the systems essentially are of a two-dimensional character. The decisive variation of the four variables (i.e. *V*, *m*, *h*, *n*) may then take place on a two-dimensional surface in the four-dimensional variable space. Hence, a model with reduction of variables (as is done in e.g. Fitzhugh-Nagumo and Morris-Lecar models [20], [56]) can give a relatively good description of an excitable membrane. The emergence of a limit cycle following a stability loss can under these circumstances be understood by the Poincaré-Bendixson theorem [57], since the system remains in a finite domain on a curved plane in the phase space. That the system remains in a finite domain is obvious from analysing the variables; the membrane potential *V* is limited by the reversal potential of Na^+^ and K^+^, as well as by the capacitive properties, and the gating parameters are limited by the values 0 and 1.

Particularly, the two-dimensional character of the models eliminates more complex types of solutions, such as irregular, “chaotic” solutions or oscillations with two separate frequencies. Nevertheless, local and highly unstable chaos has been reported in a Hodgkin-Huxley system [36], why the models are unlikely to be two-dimensional in the whole parameter space. A more stable chaos seems to require that more voltage-dependent ion channels are added to the model. We thus added two artificial ion channels to the hippocampal model and found chaotic firing (Figure S2). A rather extensive search for chaotic firing in models with just one added ion channel gave no positive results.

## Methods

### Time evolution of the membrane potential

The time evolution of the membrane potential (V) was calculated by solving the following equation (derived from Equation 1) numerically:
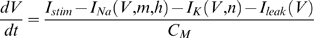
(3)where *I_Na_* and *I_K_* are functions of the activation parameters *m* and *n*, and the inactivation parameter *h*. *I_leak_* is given by
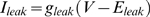
(4)
*I_Na_* and *I_K_* for the hippocampal and the myelinated axon model are described by the following expressions, based on the permeability concept of Goldman [26] and Hodgkin and Katz [27]:
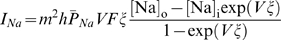
(5)and
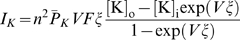
(6)where 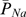 and 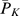 denote the Na and K permeabilities when all Na and K channels are open, and thus represent the Na and K channel densities. *R*, *T* and *F* denote the gas constant, the thermodynamic temperature and the Faraday constant, respectively, and define 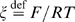. [*Na*]*_o_*, [*Na*]*_i_*, [*K*]*_o_* and [*K*]*_i_* are the external and internal concentrations of Na and K ions. The parameter values for the three models are listed in Table 3.

**Table 3:** Parameter Values for the Models.

Parameter	Value		
	Hippocampal neuron	Myelinated axon	Squid axon
T	295K	295K	
*V_rest_*	−70 mV	−70 mV	−60 mV
[Na]i	14 mM	14 mM	
[Na]o	114.5 mM	114.5 mM	
[K]i	120 mM	120 mM	
[K]o	2.5 mM	2.5 mM	
*E_Na_*			55 mV
*E_K_*			−72 mV
*E_Leak_*	−70 mV	−70 mV	−49.5
*g_Leak_*	2.32 S/m^2^	303 S/m^2^	3.0 S/m^2^
*C_m_*	70 mF/m^2^	20 mF/m^2^	10 mF/m^2^

V_1/2_ is the midpoint value and s the slope value of the activation (*m_∞_^2^*−*V* or *m_∞_^3^*−*V*, and *n_∞_^2^*−*V* or *n_∞_^4^*−*V*) or the inactivation (*h_∞_*−*V*) curves (in mV), as fitted to the equation 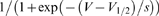. The voltage-dependent parameters in the squid axon model are based on the assumption that the resting potential is −60 mV [58].

For the squid axon model we use the original expressions by Hodgkin and Huxley [23] based on the conductance concept:
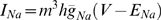
(7)and
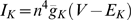
(8)where 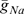 and 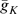 denote the Na and K conductances when all Na and K channels are open, thus representing Na and K channel densities.

The activation and inactivation parameters (*m*, *h* and *n*) are in all three models described by their time derivatives:
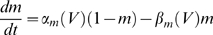
(9)

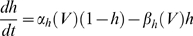
(10)

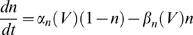
(11)where *α_i_* and *β_i_* denote rate functions. For the hippocampal neuron model they are defined as follows:
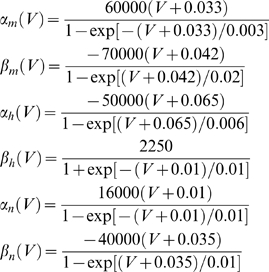
(12)For the myelinated axon model the rate functions are defined as:
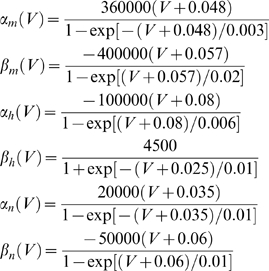
(13)For the squid axon model the rate functions are defined as:
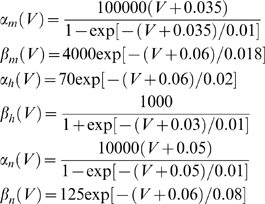
(14)


### Stability analysis of the steady-state values

The stability analysis of the differential equations was performed as briefly described by Århem et al. [11]. The stationary potentials can be calculated with the expression for the gating parameters (*m*, *n* and *h*) at steady state together with Equation 3. The time derivates of the gating parameters are zero at stationary potentials, and hence the stationary values of the parameters (denoted *m_∞_* etc) become:
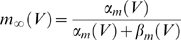
(15)

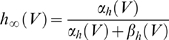
(16)

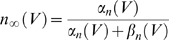
(17)Introducing these expressions into Equation 3, we obtain the following equation, the roots of which yield the stationary potentials (*V_s_*):
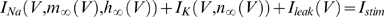
(18)This equation can be solved numerically and always yields at least one *V_s_*. However, if the Na channel density (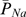 or 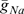) is large enough, the equation can for a defined stimulation interval give three equilibrium points, a requisite for the system to provide a saddle-node bifurcation (see Figure 3).

We investigated the character of the equilibrium points, i.e. r*(*V,m_∞_(V),h_∞_(V),n_∞_(V)*), when the stimulation current (*I_stim_*) and the permeability or conductance parameters (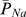 and 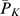 or 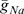 and 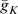) representing the density of Na and K channels, were varied. This was done by linearizing the differential equations close to r* and by solving the characteristic equation
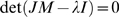
(19)where 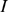 denotes the identity matrix, and *JM* the Jacobian matrix
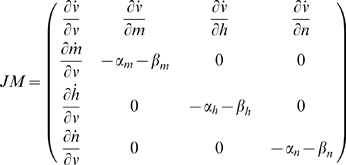
(20)where 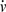, 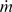, 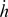 and 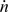 denote the time derivatives of the parameters. The solution to Equation 18 are the four eigenvalues *λ_i_* (i = 1, 2, 3 or 4) yielding an approximate time evolution of the system. Hence any perturbation **δr** around the equilibrium point r* can be written as
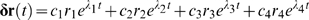
(21)where *c_i_* (i = 1, 2, 3 or 4) depends on initial conditions and *r_i_* (i = 1, 2, 3 or 4) is the associated eigenvector. Two of the eigenvalues are in the present system (here called *λ_3_* and *λ_4_*) always real and negative. Consequently the remaining two eigenvalues determine the character of the *V_s_* (see Table 4); the two negative eigenvalues will cause its associated terms in Equation 20 to decay to zero. Hence, Equation 20 can be approximated as
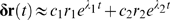
(22)


**Table 4:** Characterization of Stationary Points Based on the Eigenvalues from Equation 15.

Character	Eigenvalues	Applies to
Stable spiral	*λ_1,2_* = −*a*±*bi*	*V_s1_* and *V_s3_*
Andronov-Hopf bifurcation	*λ_1,2_* = *0*±*bi*	*V_s1_* and *V_s3_*
Unstable spiral	*λ_1,2_* = *a*±*bi*	*V_s1_* and *V_s3_*
Stable node	*λ_1_* = *−a_1_*, *λ_2_* = *−a_2_*	*V_s1_* and *V_s3_*
Saddle node	*λ_1_* = *a_1_*, *λ_2_* = *−a_2_*	*V_s2_*
Saddle-node bifurcation	*λ_1_* = *0*, *λ_2_* = −*a_2_*	*Coalescence of V_s1_ and V_s2_*
Bogdanov-Takens bifurcation	*λ_1,2_* = *0*	*Coalescence of V_s1_ and V_s2_*

Two of the four eigenvalues are always real and negative and are not included in the table. *a* and *b* are real positive numbers.

If *λ_1_* and *λ_2_* are a complex conjugated pair (*λ_1,2_* = *a*±*bi*), one can rewrite the equation, using Euler's formula, as
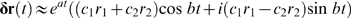
(23)why *b/2π* correlates with the firing frequency.

All computations were done in custom software written in *Mathematica* 6.0.2 (Wolfram Research, Inc.) on a 64-bit IBM compatible computer. All values are given in SI-units.

## Supporting Information

Figure S1 Firing frequency as a function of Na channel density in a rat axon model. The firing frequency at threshold is plotted against Na permeability constant. For Na permeabilities less than 59µm/s the axon model does not show any repetitive firing. The curve is calculated from the equations described in Text S1, based on the voltage-clamp analysis by Brismar [1]. Since this axon exclusively comprises Na and leak channels (it lacks K channels), the curve implies that this rat axon, in agreement with the Xenopus and the squid axon discussed in the main text, exclusively shows Type 2 dynamics for all channel densities. The *I_ss_-V* relationship is non-monotonic (N-shaped) for values of higher than 166 µm/s.
(0.54 MB EPS)

Figure S2 Chaotic firing in a modified hippocampal neuron model. Time course and phase-plot of a modified hippocampal neuron model, including an extra Na and an extra K channel. The figure depicts data from an extensive search for parameter values that give the model chaos like dynamics (i.e. aperiodic appearance over the time scale simulated). The extra channes were assumed to be described by the same kinetics as the hippocampal channels, i.e. by equations 4, 5 and 6 in the Methods section. The rate functions of the extra channels were calculated from equation 12 with the numerical values altered randomly ±50%. The permeability constants ( and in equations 5 and 6) were altered randomly in the range 0–100 µm/s, and the stimulating current in the range 0–1000 mA/m^2^. The search was performed using custom written software in Mathematica 6.0.2. Out of about 100.000 trials, eight parameter combinations yielded chaos like firing. When a similar search, equally extensive (100 000 trials), was performed with an hippocampal model comprising only one extra channel (Na or K), no positive results were found. The results seem to suggest that at least four channels (two Na and two K channels), and thus a seven-dimensional system, are required to give neuron models of the type studied here chaotic dynamics.
(1.63 MB EPS)

Text S1 A model of the myelinated rat axon
(0.07 MB PDF)
