# Supplementary material for: Ion Channel Density Regulates Switches between Regular and Fast Spiking in Soma but Not in Axons
Source: PLoS Comput Biol. 2010 Apr 22;6(4):e1000753. doi: 10.1371/journal.pcbi.1000753 (PMC2858683; doi:10.1371/journal.pcbi.1000753)
Supplement: Text S1 — A model of the myelinated rat axon (0.07 MB PDF) [file pcbi.1000753.s003.pdf]

A MODEL OF OF THE MYELINATED RAT AXON

H. Zeberg*, C. Blomberg† and P. Århem*
* The Nobel Institute for Neurophysiology, Department of Neuroscience, Karolinska Institutet, SE-17177 Stockholm, Sweden;
and † Department of Theoretical Physics, KTH-Albanova, SE-62016 Stockholm, Sweden

This text aims to describe the electrical behaviour of a model nerve fibre whose properties are based on the results of a voltage clamp study of myelinated axons from the sciatic nerve of Sprague-Dawley rats (*Rattus Norvegicus*)[1].

The model is given by the following coupled differential equations

$$\frac{dV}{dt} = \frac{I_{stim} - I_{Na}(V,m,h) - I_{leak}(V)}{C} \tag{1}$$

$$\frac{dm}{dt} = \alpha_m(V)(1-m) - \beta_m(V)m \tag{2}$$

$$\frac{dh}{dt} = \alpha_h(V)(1-h) - \beta_h(V)h \tag{3}$$

where

$$I_{Na} = m^2 h \overline{P}_{Na} V F \xi \frac{[Na]_e - [Na]_i \exp(V\xi)}{1-\exp(V\xi)} \tag{4}$$

with $\xi \stackrel{\text{def}}{=} F/RT$ (F, R and T having their usual thermodynamic meaning) and

$$I_{leak} = g_{leak}(V - E_{leak}). \tag{5}$$

$\alpha_i(V)$ and $\beta_i(V)$ denote rate functions for the activation ($i = m$) and inactivation ($i = h$) variables and are of the form

$$f_i(V) = A_i \frac{V + B_i}{1 - \exp(C_i(V + B_i))} \tag{6}$$

where the constants A, B and C can be found in the table below.

$f_i(V)$	A	B	C
$\alpha_m(V)$	720 000	0.048	-333
$\beta_m(V)$	-400 000	0.057	50
$\alpha_h(V)$	-29 000	0.07	200
$\beta_h(V)$	42 000	0.058	128

A, B and C have the units $V^{-1}s^{-1}$, V and V^{-1} respectively. The remaining parameter values are given in the following table.

Parameter		Value
Absolute temperature	T	294°K
Internal Na concentration	$[Na]_i$	17 mM
External Na concentration	$[Na]_o$	147 mM
Membrane capacitance	C_m	20 mF/m^2
Na permeability constant	$\overline{P}_{Na}$	80 μm/s
Leak reversal potential	E_{leak}	-80 mV
Leak conductance	g_{leak}	1.3 kS/m^2

The parameter values for the equations were chosen to describe the currents at 24°C. The rate functions for the inactivation parameters $\alpha_h(V)$ and $\beta_h(V)$ used were those given by Brismar [1], while the activation parameters $\alpha_m(V)$ and $\beta_m(V)$ were estimated by fitting calculated curves to the recorded currents. The formalism used by Frankenhaeuser and Huxley [2] to describe the Na currents in axons of the African clawed frog (*Xenopus leavis*), comprising the permeability concept and an exponent of two for the activation, was found to well describe also the rat axon Na current. Also the capacitance value was estimated to be equal in myelinated frog and rat axons. The model utilizes SI units throughout.

References

1. Brismar T (1980) Potential clamp analysis of membrane currents in rat myelinated nerve fibres. J Physiol 298: 171-184.
2. Frankenhaeuser B, Huxley AF (1964) The Action Potential in the Myelinated Nerve Fiber of Xenopus Laevis as Computed on the Basis of Voltage Clamp Data. J Physiol 171: 302-315.
